# Reversible Contraceptive Potential of FDA Approved Excipient N, N-Dimethylacetamide in Male Rats

**DOI:** 10.3389/fphys.2020.601084

**Published:** 2020-11-10

**Authors:** Nupur Khera, Chafik Ghayor, Anna K. Lindholm, Ekaterina Pavlova, Nina Atanassova, Franz E. Weber

**Affiliations:** ^1^Oral Biotechnology and Bioengineering, Center of Dental Medicine, University of Zurich, Zurich, Switzerland; ^2^Zurich Center for Integrative Human Physiology (ZIHP), University of Zurich, Zurich, Switzerland; ^3^Department of Evolutionary Biology and Environmental Studies, University of Zurich, Zurich, Switzerland; ^4^Institute of Experimental Morphology, Pathology and Anthropology with Museum, Bulgarian Academy of Sciences, Sofia, Bulgaria; ^5^Center for Applied Biotechnology and Molecular Medicine, University of Zurich, Zurich, Switzerland

**Keywords:** male contraception, bromodomain (BRD), reproduction, excipient, rat, BET inhibitor

## Abstract

Development of an effective male contraceptive agent remains a challenge. The present study evaluates the potential of N, N-Dimethylacetamide (DMA), a FDA approved excipient as a male contraceptive agent. Male Sprague Dawley rats injected with DMA for a period of 8 weeks (one injection per week) showed a significant alteration of reproductive parameters. Furthermore, DMA treated animals showed complete infertility in a dose dependent manner, as no pups were born despite proper mating between females and DMA treated males. However, stopping the DMA treatment for a period of 8 weeks (after the initial treatment) restored the reproductive parameters to normal. Moreover, the fertility was resumed to normal as pups were born in the groups where DMA treatment was halted after initial DMA treatment. All these changes had no effect on the level of reproductive hormones FSH, LH and testosterone. Taken together, our results indicate that DMA acts in a reversible and non-hormonal manner to achieve contraception in rats. Therefore, repurposing the use of DMA could lead in a short time to an inexpensive and safer male contraceptive option.

## Introduction

The rate of unwanted pregnancies is globally increasing. About 40% of all pregnancies were unintended in 2012 (Sedgh et al., 2014). This high rate is due to barriers for women to access contraceptives and insufficient knowledge of available contraceptive methods. Certain fraction of women who take the female contraceptive pills have to quit it due to development of high risk of venous thromboembolism, breast cancer and other side effects (McDaid et al., 2017). Another major and often neglected reason leading to unintended pregnancies is the lack of a reliable male contraceptive method (Plana, 2017), leaving couples with limited contraceptive options. No successful male contraceptive agent or method has been developed since condoms in the 18th century and vasectomy in the 19th century (Drake et al., 1999; Khan et al., 2013). A certain proportion of men face difficulties while using condoms as use of latex condoms can lead to allergic reactions and skin irritation. For some men the use of condoms is associated with a decrease in sexual pleasure (Newby et al., 2013; Caminati et al., 2019). Vasectomy, by contrast, requires a surgery under local anesthesia where the vas deference is surgically interrupted bilaterally through a small incision in the scrotum. This is the best choice for couples who do not want further children. Although the process is surgically reversible, the pregnancy rates after reversal vary from 50–75% depending on the time between the vasectomy and the reversal surgery (Belker et al., 1991; Heidenreich et al., 1994). Therefore, vasectomy cannot be labeled as a reliable reversible contraceptive method and an urgent need for a potent, reliable and reversible male contraceptive agent persists.

The sensitivity of the spermatogenesis process to hormonal regulation was long believed to be a promising route for the development of a male contraceptive (Wang and Swerdloff, 2010). To this end, Testosterone and its analogs have been injected to signal the hypothalamus to stop producing GnRH and thus inhibiting intra testicular testosterone release. Even though this approach reduces sperm counts, the method was never 100% effective (Contraceptive efficacy of testosterone-induced azoospermia in normal men. World Health Organization Task Force on methods for the regulation of male fertility, 1990). Although many novel hormonal molecules are in clinical trials, other non-endocrine approaches are also considered and would be more favorable (Thirumalai and Page, 2019). Multiple genes and proteins involved in sperm production and transport are attractive targets. If the function of any of these genes or proteins could be blocked, it would become a potential target for male contraception purposes.

In recent years, several inhibitors for Bromodomain and Extra-Terminal motif (BET) proteins were synthesized and their epigenetic activities on diverse tissues analyzed in depth. The BET family of bromodomain proteins include BRD2, BRD3, BRD4, and BRDT, which are expressed at distinct stages during spermatogenesis (Berkovits and Wolgemuth, 2013). It was shown that inhibition of BRDT by small BET inhibitor JQ1 leads to reversible contraception in male mice (Matzuk et al., 2012). N, N-Dimethylacetamide (DMA), an FDA approved solvent, acts as a low affinity inhibitor for BRD2 and BRD4 (Ghayor et al., 2017). N, N-Dimethylacetamide is a widely used pharmaceutical agent, regularly injected in humans as a solvent facilitating the application of insoluble drugs (Hassan, 1999; Hempel et al., 2007). Ideally, an excipient should not possess any biological activity. However, in a recent study, we have shown that DMA affects spermatogenesis and leads to infertility by acting non-hormonally and post-meiotically (Khera et al., 2020).

In the current study, we examined the effects of DMA treatment on spermatogenesis and infertility *in vivo* with emphasis on a possible recovery once the treatment is halted. Since the process of spermatogenesis is conserved over species, results from our rat model could be indicative for a possible use of DMA as a non-hormonal, post meiotically acting and reversible male contraceptive in humans.

## Materials and Methods

### Animals Experiments

All animal procedures were according to the ARRIVE guidelines approved by the Animal Ethics Committee of the local authorities (Veterinäramt, Canton Zurich, project codes: 40/2012 (approved on the April 2, 2012) and 068/2015 (approved on the July 31, 2015), and follow the EU Directive 2010/63/EU for animals. Male Sprague Dawley rats (age: 6 weeks old) were ordered from Charles River Laboratories. Three animals were pooled in one cage and were acclimatized to the animal facility environment for a period of two weeks. After two weeks, each of the animals were weighed and randomized. The animals were divided into two groups: PBS group and the DMA treated group. They were injected as previously described (Khera et al., 2020). Volume of DMA to be injected was one third of the LD50 (862 mg/kg) (Bartsch et al., 1976). This dose was found to be effective in our previous studies of DMA on rats (Ghayor et al., 2017). Animals were injected once per week, intra-peritoneally for eight consecutive weeks, which is equivalent to one spermatogenesis cycle in rats. In addition, a pilot study with *n* = 3 showed a significant decrease in sperm count and alteration of seminiferous epithelium (data not shown) after 8 weeks (one injection per week), therefore the chosen dose was continued. At the end of 8 weeks, the treatment was stopped for another 8 weeks. The animals were analyzed three times weekly for any abnormalities. At the end of a total of 16 weeks (8 weeks of DMA/PBS treatment plus 8 weeks of stopping the treatment) the animals were sacrificed, and their reproductive parameters were analyzed.

For the breeding experiments, one male was mated with one female and the pups were analyzed at subsequent time intervals (explained in detail in results section).

### Hematoxylin and Eosin Staining

The testis and epididymis were collected in bouin’s solution. Liver was collected in formalin. Histological sections and Hematoxylin and eosin (H&E) stained slides were performed as previously described (Khera et al., 2020). The sections were analyzed blindly by two independent experimenters.

### Measurement of Area of Seminiferous Tubules

Hematoxylin and eosin stained testis slides were used to calculate the area of seminiferous tubules. For each sample the area of twenty seminiferous tubules was randomly calculated using ImageJ software.

### qPCR Analysis

RNA was extracted using TRIzol reagent (Invitrogen) and reverse transcribed into cDNA using iScript reverse transcription supermix (Bio-Rad). RT-qPCR was performed using gene-specific primers (Supplementary Table) with SsoAdvanced universal SYBR green supermix and the CFX Connect real-time system (Bio-Rad) using beta actin as the housekeeping gene.

### Computer Assisted Sperm Analysis

The left epididymis was collected in HTF medium (Irvine Scientific, Article number 90126). Caudal epididymis was carefully cut and punctured using a syringe needle. It was then incubated in pre warmed HTF medium for 10 min at 37°C. The spermatozoa were allowed to swim out in the medium and then collected in a 1.5 mL Eppendorf tube. A Computer Assisted Sperm Analyser (Hamilton) was used to measure the sperm count and percentage motility.

### Cell Counting for Spermatids

For histological analyses, Bouin’s fixed, paraffin embedded tissue sections were stained with H&E. Testicular cell composition was estimated using standard stereological techniques involving point counting of cell nuclei to determine the nuclear volume per testis of round, elongated and total spermatids, as previously described (Welsh et al., 2009). In brief, cross sections of testis were examined using 63× objective fitted to Zeiss AxioScope A1 and a 121-point eyepiece graticule (Leica Microsystems, Wetzlar, Germany). Applying a systematic sampling pattern from a random starting point, 32 microscopic fields (3872 points) were counted for each animal. Points falling over Sertoli cell or germ cell nuclei, seminiferous epithelium, interstitium, seminiferous tubule lumen were scored, and they were expressed as relative (%) volume per testis. For round, elongated and total spermatids, the values for percent nuclear volume were converted to absolute nuclear volumes per testis by reference to testis volume (weight) because shrinkage was minimal. The results were represented as percentage change in DMA groups compared to the PBS group.

Statistical Analysis: Statistical analyses was performed with GraphPad Prism 7. Results were expressed as the mean ± SD and were compared by Student’s *t*-test. All results with p < 0.05 were considered significant.

## Results

### DMA Reversibly Influences the Reproductive Parameters

**FIGURE 1:**
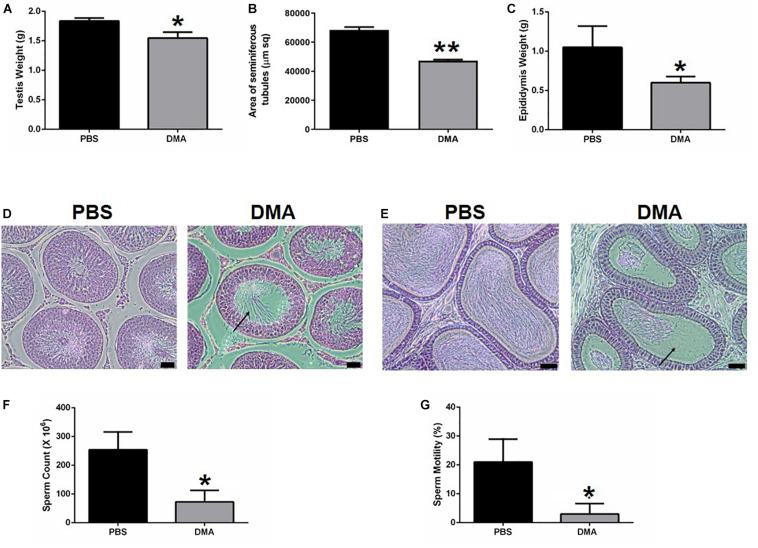
Effect of DMA versus PBA treatment on reproductive parameters (*n* = 3). Effect of DMA treatment on **(A)** testis weight, **(B)** area of seminiferous tubules, and **(C)** epididymis weight. **(D)** Histological analysis of testicular sections. Arrow indicated empty seminiferous tubule lumen (scale bar = 100 μm). **(E)** Histological analysis of the Epididymis. Arrow indicates absence of sperm (scale bar = 100μm). **(F)** Sperm count and **(G)** percentage motility of PBS and DMA treated animals. Data represents mean ± SD (*p < 0.05, **p < 0.01, obtained by two tailed student *t*-test).

Previously we have shown that DMA treatment causes significant changes in reproductive parameters by decreasing the spermatid counts (Khera et al., 2020). To determine whether DMA discontinuation could reverse these parameters, we performed another independent experiment. Eight male rats were treated with either PBS or DMA for 8 weeks after which three animals per group were sacrificed to ensure that the reproductive parameters were decreasing as previously reported (Khera et al., 2020). The other five animals were kept without any further injections. The animals that were sacrificed after 8 weeks of DMA treatment showed a significant decrease in testis and epididymis weight. Moreover, distortions in seminiferous tubules and a reduced sperm count and motility were observed. A concomitant distortion of epididymal histology was evident (Figure 1D). All these results on reproductive parameters (Figure 1) confirmed the negative effect of DMA on reproductive parameters of animals as previously reported (Khera et al., 2020).

**FIGURE 2:**
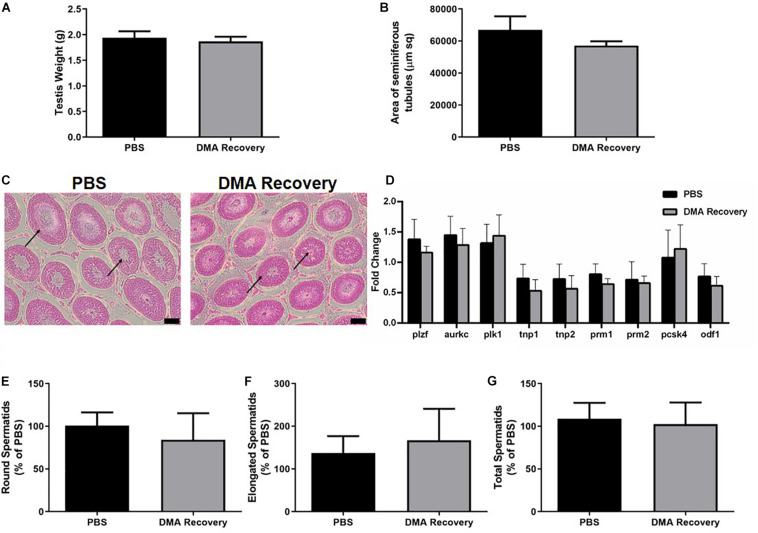
Effect of stopping DMA treatment on reproductive parameters. Effect of stopping the DMA treatment on **(A)** testis weight (*n* = 5) and **(B)** area of seminiferous tubules (*n* = 3). **(C)** Histological analysis of testicular sections (*n* = 3). Arrows indicate normal seminiferous tubule lumen in both PBS and DMA recovery groups (scale bar = 100 μm). **(D)** Quantitative RT-PCR analysis of genes expressed at different stages of sperm maturation (*n* = 3). Percentage of **(E)** round spermatids, **(F)** elongated spermatids, and **(G)** total spermatids in DMA recovery group compared to the PBS group (*n* = 3). Data represents mean ± SD.

The other five animals were then housed for the next 8 weeks without any further treatment. At the end of 16 weeks (8 weeks of injections and 8 weeks of no injections) the animals were sacrificed, and their reproductive parameters analyzed. There was no significant difference in the testis weight and the area of seminiferous tubules comparing DMA recovery (where DMA treatment was stopped) and PBS controls (Figures 2A,B). Moreover, the seminiferous epithelium of animals from the DMA recovery group was indistinguishable from that of PBS controls (Figure 2C). The level of gene expression of markers for spermatogonia (plzf), spermatocyte (aurkc, plk1) and spermatids (tnp1, tnp2, prm1, prm2, pcsk4, odf1) was in the same range and insignificantly different for DMA recovery and PBS treated groups (Figure 2D). Previously, without a recovery phase of 8 weeks, DMA treatment lead to a significant decrease in the number of elongated and total spermatids (Khera et al., 2020). Next, we did the cell counting for round, elongated and total spermatids for DMA recovery and PBS groups. Germ cell counting results showed no differences in number of spermatids thus confirming that testicular features returned to normalcy after stopping the DMA treatment for 8 weeks (Figures 2E–G).

**FIGURE 3:**
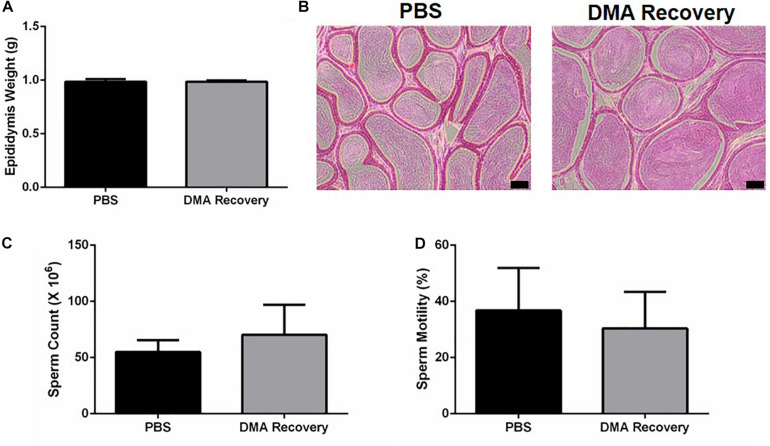
Effect of stopping the DMA treatment on epididymal parameters. **(A)** Epididymis weight (*n* = 5). **(B)** Histological analysis of the Epididymis (scale bar = 100 μm) (*n* = 3) **(C)** sperm count and **(D)** percentage motility of PBS and DMA Recovery group animals (*n* = 5). Data represents mean ± SD.

Since testicular functions appeared completely normal, we next wanted to see the effect of stopping the DMA treatment on the epididymal parameters. Epididymal weight and histology was indistinguishable in both the groups (Figures 3A,B). No significant changes were observed in sperm count and motility (Figures 3C,D and Supplementary videos, Video 1: PBS, Video 2: DMA Recovery). Moreover, no significant changes were observed in any of the computer assisted sperm analysis (CASA) parameters listed in Table 1.

**TABLE 1:** Measurement of CASA parameters in epididymal sperm of PBS and DMA Recovery groups.

Parameter			PBS	DMA Recovery
Path velocity	VAP	μm/s	165.68 ± 15.4	173.08 ± 13.6
Prog. velocity	VSL	μm/s	106.82 ± 13.97	112.94 ± 12.0
Track speed	VCL	μm/s	334.68 ± 23.3	340.22 ± 25.5
Lateral amplitude	ALH	μm	16.8 ± 1.15	16.48 ± 1.90
Beat frequency	BCF	Hz	28.5 ± 1.50	26.84 ± 3.26
Straightness	STR	%	62.67 ± 3.50	62.6 ± 3.31
Linearity	LIN	%	34.16 ± 1.94	35 ± 3.90
Elongation		%	25.34 ± 2.87	25.4 ± 4.71
Area		μm sq	242.93 ± 67.95	199.54 ± 63.06

*Data represent mean ± SD (*n* = 5).*

### DMA Causes Infertility in Rats

**FIGURE 4:**
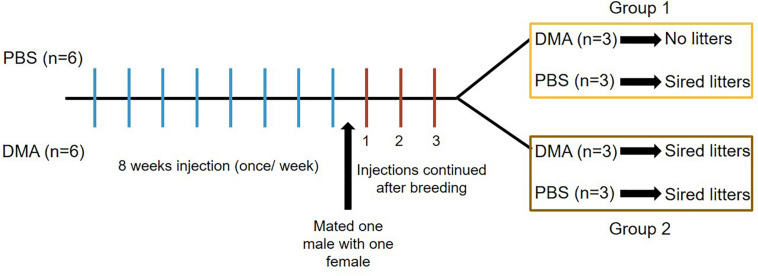
Diagrammatic representation of breeding experiments. Three weeks after the start of breeding, Group 1 animals received either PBS or DMA injections once/week. Group 2 animals received either PBS or DMA injections twice/week. 6 PBS animals were randomly divided into three each for group 1 and group 2.

Since 8 weeks of recovery from DMA treatment was able to reverse the damaging effect on reproductive parameters, we further determined the effect of DMA treatment on fertility of male rats. For this, we treated a new set of male rats with PBS or DMA (once/week) for a period of 8 weeks and then subjected these animals to a mating experiment during which one treated male was caged with one untreated female. The injections were continued during the mating experiments and pups were analyzed at subsequent time intervals. After a period of three weeks of breeding, all six PBS-treated animals sired litters. However, in the DMA treated group, three animals sired litters and the other three did not. We, therefore, regrouped the DMA treated animals according to sensitivity, where group 1 comprised of animals sensitive to 8 weeks of once per week DMA treatment (i.e., siring no litters) and group 2 consisting of animals insensitive to 8 weeks of once per week DMA treatment (i.e., still fertile). The six PBS controls, who sired litters, were randomly distributed into the two groups (3 PBS animals in group 1 and 3 PBS animal in group 2) (Figure 4).

**FIGURE 5:**
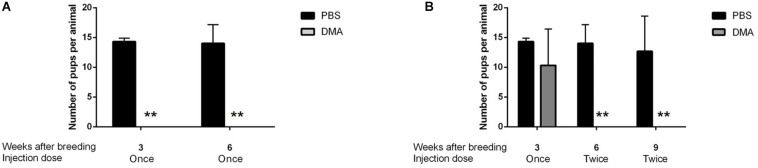
Number of pups born during DMA treatment. **(A)** Number of pups born per animal in PBS and DMA treated animals after 8 weeks of initial treatment plus 6 weeks of continuing treatment while breeding: Group1 (*n* = 3). **(B)** Number of pups born per animal in PBS and DMA treated animals after 8 weeks of initial treatment plus 3 weeks of once per week plus 6 weeks of twice per week continuing treatment while breeding: Group2 (*n* = 3). Data represents mean ± SD (**p < 0.01, obtained by two tailed student *t*-test).

During the following 3 and 6 weeks of continuing once per week DMA treatment, group 1 animals appeared to be sterile since the females caged with the DMA treated animals did not produce pups (Figure 5A).

For group 2, we then increased DMA treatment to twice a week and continued mating. After increasing the injection frequency to twice a week over a period of 3 weeks, the animals of group 2 turned completely infertile for the next six weeks, where they were housed with female rats (Figure 5B). Therefore, Group 2 animals were characterized to be completely infertile during 6 weeks of twice/week treatment. Thus, depending on individual variability of each of the DMA treated males, a complete contraceptive effect can be reached in rats in a DMA concentration dependent manner.

### DMA Causes Reversible Infertility in Rats

**FIGURE 6:**
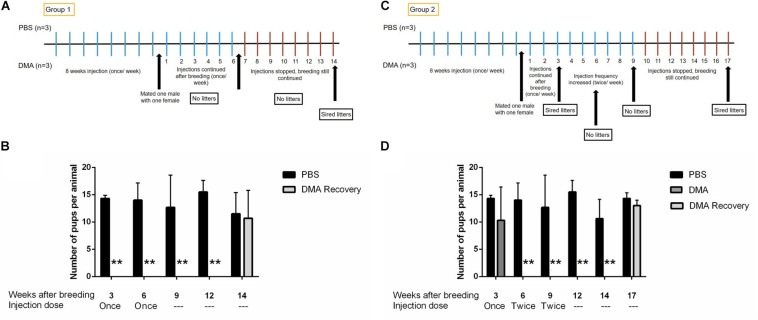
Number of pups born after stopping DMA treatment. Number of pups born per animal in PBS and DMA treated animals after 3, 6, and 8 weeks of stopping the treatment (*n* = 3). **(A)** Diagrammatic representation of experimental protocol: Group 1. **(B)** The animals that showed complete infertility at a dose for once/week DMA treatment, produced pups 8 weeks after the treatment was stopped: Group1 (*n* = 3). **(C)** Diagrammatic representation of experimental protocol: Group 2. **(D)** The animals that showed complete infertility after escalating the dose to twice per week showed complete fertility after 8 weeks of stopping the DMA treatment: Group2 (*n* = 3). Data represents mean ± SD (**p < 0.01, obtained by two tailed student *t*-test).

To examine the reversibility of DMA induced contraception, we assessed if fertility was restored upon cessation of DMA treatment. For both, Group 1 and Group 2, the fertility was regained 8 weeks after stopping the once or twice a week DMA treatment. The pups born in both the groups were, in terms of number, statistically similar to ones born from PBS treated control animals (Figure 6). The offspring appeared to be morphologically similar in both groups and developed normally for one week of their life. No abnormalities were observed in the major organs like brain, liver and heart as examined by MRI analysis (Supplementary Figure 1).

### Effect of Stopping the DMA Treatment of Hormonal Levels

**FIGURE 7:**
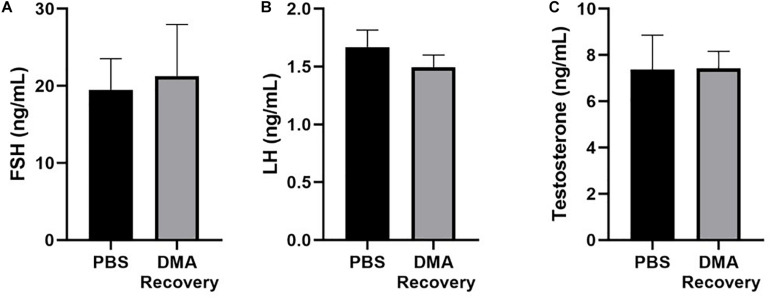
Serological analysis of hormonal levels. **(A)** Serum FSH, **(B)** serum LH, and **(C)** serum testosterone levels in PBS and DMA recovery groups (*n* = 3). Data represents mean ± SD.

Our previous data has shown that DMA treatment had no effect on levels of sexual hormones (Khera et al., 2020). To study if stopping DMA treatment maintained hormone levels, we analyzed the serum levels of testosterone, follicular stimulating hormone (FSH) and luteinizing hormone (LH) and found no significant changes in the hormonal levels after stopping DMA treatment (Figure 7).

## Discussion

Development of a reversible, non-hormonal and safe male contraceptive remains a challenge. The major reason for this lies in the physiological differences in the male and female reproductive systems. A female contraceptive should suppress the fertilization of one ovum per menstrual cycle. In contrast, a male contraceptive has the obligation of blocking 15–200 million sperms/mL of ejaculate. Moreover, the spermatogenesis cycle lasts for 74 days, therefore, contraceptives should be regularly given for a period of at least 5–8 weeks before a wanted contraceptive state can be reached (Griswold, 2016). Although female contraceptives are very effective at preventing unintended pregnancies, many women cannot use them because of various side effects or health conditions (National Research Council Committee on Population, 1989).

Previous studies from our lab and the current study has focused on the use of DMA as a non-hormonal and reversible male contraceptive agent in rats. Administration of DMA once a week for 8 weeks (equivalent to one spermatogenic cycle in rats) negatively affects the reproductive parameters. Additional injections for 3 weeks for DMA sensitive rats (Group 1) or a further increased frequency for insensitive rats (Group 2) lead to complete infertility in rats with no pups being born. In addition, no changes were found in the sexual behavior of the treated rats, which was also confirmed by the absence of changes in serum testosterone levels. Moreover, vaginal smear experiments showed the presence of spermatozoa even when no pups were being born (Supplementary Figure 2).

The effect of DMA is completely reversible as pups were born eight weeks after stopping the treatment in both the experimental groups (Group1 and Group2) where once or twice a week treatment with DMA was initially needed for male sterility. The gestation period in rats is three weeks and since pups were born after 8 weeks of stopping the treatment, implied that the females were pregnant after 5 weeks of stopping the treatment. These results were also consistent with the fact that DMA affects the post meiotic stages of spermatogenesis. We also observed spermatozoa in the vagina of female rats in a vaginal smear experiment, five weeks after starting the mating experiments (Supplementary Figure 2). Thus, DMA acts in a post-meiotic manner to achieve reversible contraception in rats.

Previously, Bromodomain inhibitors with very high affinity to BET proteins, like JQ1, have been used to achieve male contraception in mice. Its male contraceptive effect was attributed to the inhibition of the testis specific BRDT protein (Matzuk et al., 2012). BRDT is expressed during pachytene spermatocyte, diplotene spermatocyte, and round spermatid stages (Shang et al., 2007). Although JQ1 shows promising results, further research has revealed that JQ1 is not well tolerated in mice. At higher doses, JQ1 causes significant decrease in body weight (Lee et al., 2016). Our results show that DMA treatment and recovery does not alter the body weights of animals compared to the PBS controls (Supplementary Figure 3). Since JQ1 causes severe side effects, other BET inhibitors must be designed specifically for male contraception. In contrast to JQ1, DMA is a low affinity bromodomain inhibitor (Ghayor et al., 2017) and is well tolerated in humans, and therefore used as FDA approved excipient. DMA has no effect on the number of spermatocytes or round spermatids within the seminiferous tubules, where BRDT is expressed (Khera et al., 2020). It appears to target mainly spermiogenesis, which is more dependent on BRD4 expression, indicated by compromised acrosomal structure, decreased number and gene expression for markers of total and elongated spermatids (tnp1, tnp2, prm1, prm2, pcsk4, and odf1) (Khera et al., 2020). This suggests that on the molecular level, male sterility by DMA acts via inhibition of BRD4 and not via BRDT, the main target of JQ1 in this concept.

In humans, DMA is injected as a drug solubilizer, e.g., to facilitate busulfan treatment in children during chemotherapy. The total concentration of DMA administered per regimen in a patient can be up to 105 mmoL (Oechtering et al., 2006; Trame et al., 2013). Data from children receiving the busulfan formulation with DMA revealed that DMA is not toxic since it is rapidly cleared from the body. Taken together these results indicate that DMA is safe for use in humans (Hempel et al., 2007). We observed no signs of toxicity as was evident by normal hepatic architecture of liver sections from PBS and DMA treated animals (Supplementary Figure 4). The current study showed DMA acts as a reversible and non-hormonal contraceptive for rats in a dose dependent manner. Since bromodomains are evolutionary conserved (Muller et al., 2011), DMA can act as a potential male contraceptive for humans. However, more research should be focused on finding the right concentration and a feasible application mode for human use.

DMA has very good skin penetration property. It has been used as a solvent for enhancing the penetration of compounds through the skin (Zhang et al., 2015). Therefore, future studies should be directed toward examining the contraceptive effect of DMA if applied with a topical gel. In the end, further research can lead to an improved application of DMA e.g., in the form of a slow release drug delivery DMA-depot patch.

Instead of developing a new molecule for male contraception, in the current study, we have repurposed the use of DMA that is already used as a solvent to administer drugs in humans. Taken together, our results show that DMA acts in a non-hormonal, post-meiotic and reversible manner to achieve reversible contraception in rats.

## Data Availability Statement

The raw data supporting the conclusions of this article will be made available by the authors, without undue reservation.

## Ethics Statement

The animal study was reviewed and approved by the Veterinäramt Zurich.

## Author Contributions

NK, CG, and FW designed the research and wrote the manuscript. NK performed the research. NK and AL performed the CASA experiments. EP and NA performed the germ cell counting for testis. NK analyzed the data. All authors read and approved the final manuscript.

## Conflict of Interest

The authors declare that the research was conducted in the absence of any commercial or financial relationships that could be construed as a potential conflict of interest.
