# Supplementary material for: Reversible Contraceptive Potential of FDA Approved Excipient N, N-Dimethylacetamide in Male Rats
Source: Front Physiol. 2020 Nov 10;11:601084. doi: 10.3389/fphys.2020.601084 (PMC7683412; doi:10.3389/fphys.2020.601084)
Supplement: Supplementary file 1 [file Data_Sheet_1.PDF]

Supplementary Material

Supplementary Table: List of primers

Gene	Forward	Reverse
plzf	CTCTGCAAGCGACCCCTC	GCATGGGGCTCTCTTTCCTT
aurkc	ACTTCGGGTGGTCTGTGC	AGTCCAGAGTCCCACACATTG
plk1	CGGACAAGTATGGCCTTGGT	AGTTTGGTGTGGTCCTGGAAG
prm1	GGCCAGATACCGATGCTG	CTCCGTCTGCGACATCTTC

Gene	Company	Catalog no.
tnp1	Qiagen	QT00378336
tnp2	Qiagen	QT00376992
prm2	Qiagen	QT00371273
odf1	Qiagen	QT02461340
pcsk4	Qiagen	QT00181503

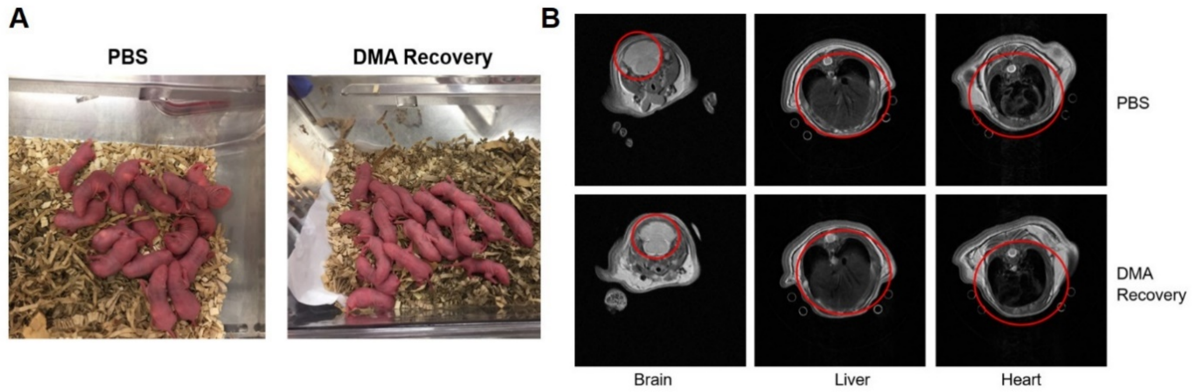

Supplementary Fig. 1: Morphology and MRI analysis of pups. Representative images of (A) Morphology of pups born in PBS and DMA recovery group at day 3 after birth, and (B) MRI analysis of pups born in PBS and DMA recovery group at day 7 after birth.

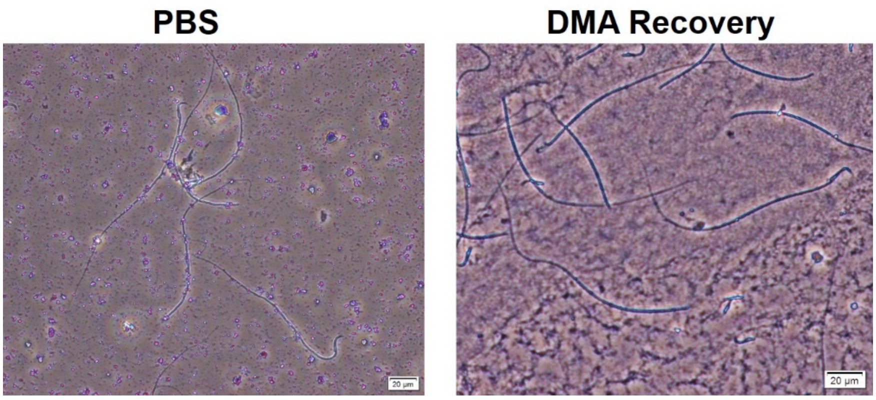

Supplementary Fig. 2: Effect of DMA on libido: Representative images of sperms prepared by smearing the vaginal fluid on slide from females mated with males (scale bar: 20µm). Presence of sperms indicate proper mating behaviour.

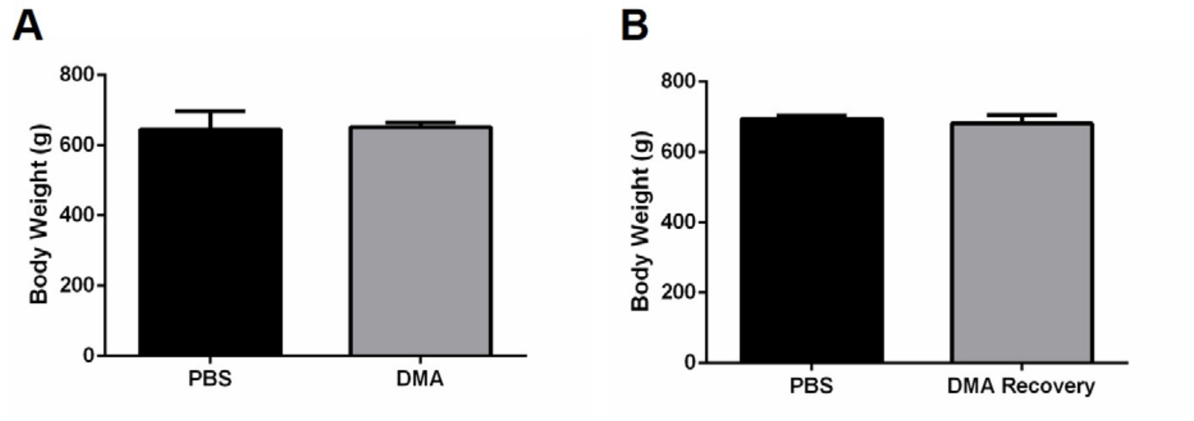

Supplementary Fig. 3: Body weight measurements. (A) Effect of DMA treatment on body weight. (B) Effect of stopping the DMA treatment on body weight. Data represents mean±sd.

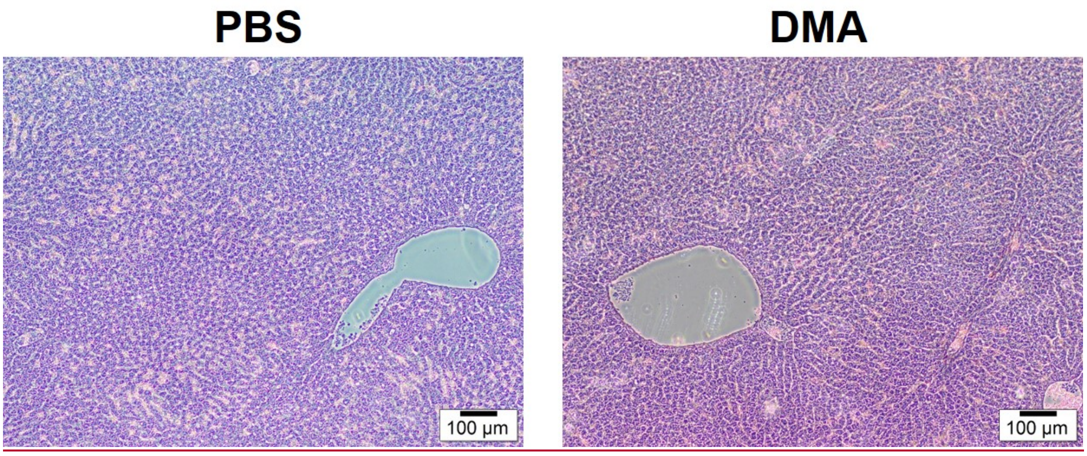

Supplementary Fig. 4: Histological analysis for toxicity. Representative images of H &E stained liver sections (scale bar: 100µm). The images show proper hepatic architecture. No areas of necrosis were observed in PBS and DMA treated groups.
